# Prevalence and factors associated with tuberculosis infection (TBI) among residents of a monastery situated in a high-TB burden area: A cross-sectional study, Sikkim, India

**DOI:** 10.1371/journal.pone.0333583

**Published:** 2025-10-13

**Authors:** Mohammad K. Siddiqui, Shagufta Khan, Rinchenla Bhutia, Vivek Nair, Ashok Rai, Nirmal Gurung, Tseten Yamphel, Peggy K. Dadul, Debya S. Kerongi, Karma Doma Bhutia, Jagat Pradhan, Kabita Khati, Sreenivas A. Nair, Shamim Mannan, Kiran K. Rade, Dinesh Gupta, Pawan Malhotra, L. Masae Kawamura, Shikha Dhawan, Asif Mohmmed

**Affiliations:** 1 Intermediate Reference Laboratory, STNM Hospital, Gangtok, Sikkim, India; 2 International Centre for Genetic Engineering and Biotechnology, New Delhi, India; 3 State TB Cell, Gangtok, Sikkim, India; 4 Stop TB Partnership, Geneva, Switzerland; 5 WHO-India Country Office, New Delhi, India; 6 Vital Strategies Board of Directors, New York, New York, United States of America; 7 Biozazen, New Delhi, India; 8 Centre for Social Integration and Borderless World, Uttar Pradesh, India; Rutgers Biomedical and Health Sciences, UNITED STATES OF AMERICA

## Abstract

**Background:**

Monasteries in India house individuals from childhood to advanced age. These congregate settings amplify tuberculosis (TB) transmission and exposure when the disease is present, especially in the high burden areas like Sikkim, India. However, the prevalence of active-TB disease (ATB), tuberculosis infection (TBI), and their associated risk factors have not been studied. The diagnosis and treatment of TBI remain a major bottleneck in eradicating TB. ATB and TBI risk among residents living in the congregate setting of monasteries in Sikkim, India, a high-TB burden area, may be high due to high-density living quarters, public interaction and their frequent travel history but has never been illustrated.

**Method:**

A cross-sectional screening of the monks and residents of Rumtek Monastery (Sikkim, India) was carried out to assess extent of ATB and TBI in a congregate setting. TrueNat MTB and GeneXpert MTB/Rif systems were utilized for ATB diagnosis, whereas QuantiFERON-TB Gold Plus (QFT-plus) Interferon-gamma release assay (IGRA) analysis was used for TBI detection. Follow-up sputum testing by TrueNat MTB was performed on IGRA-positive individuals to exclude ATB.

**Results:**

Among the 350 inhabitants of the monastery, 7% (25/350) were found to be symptomatic for TB, whereas 93% (325/350) were asymptomatic. Out of them, 189 participants, including symptomatic cases, agreed to participate in the study and were screened for TBI; however, 15 participants were excluded from the study due to result discrepancies. None of the participant were diagnosed with active tuberculosis (ATB), although, 44.2% (77/174) were found to be positive for TBI; however, none of those with TBI progressed to ATB during one year follow-up. Risk factors for TBI included: advancing age, frequent travel history, family history of TB or having contacts with TB patients and abnormal Body Mass Index (BMI) ≤18.5- ≥ 25.

**Conclusion:**

This study confirms the high prevalence of TBI among residents in the congregate setting of monasteries, and justify TB prevention strategies by targeted screening, TBI testing and preventive treatment in congregate settings of high TB burden areas.

## Introduction

Tuberculosis (TB) continues to be a public health problem worldwide. The disease is one of the most important causes of death from infectious diseases and the massive global reservoir of tuberculosis infection (TBI), ensures future disease in the human population [1]. National TB Prevalence Survey (2019–2021) estimates the prevalence of TBI in India to be 33% [2]. About 5–10% of people with TBI progress to active-TB disease (ATB) in their lifetime and the majority of them get the disease within two years of getting the latent TB infection (TBI) [3,4]. Although the treatment of ATB remains a top priority in endemic settings, this approach alone is not sufficient to achieve a steep annual reduction in incidence necessary to reach the world health organization’s (WHO) End TB Strategy targets; thus, preventive treatment to stop the progress of TBI to ATB is one of the main interventions needed to achieve TB elimination [5]. Improved attention to TBI screening and preventive therapy is crucial for the End TB Strategy for 2050 [6–8]. Frameworks towards End TB strategy focuses on low-populated areas diagnosis and treatment of LTBI [9]. Under National TB Elimination Programme (NTEP) LTBI management in India mainly focuses on high risk groups such as house hold contacts of ATB patients, immunocompromised and health-care workers; NTEP also recommends TB preventive treatment (TPT) regimes for TBI.

Sikkim is a state in the north-eastern region of India, a part of Eastern Himalaya and is notable for its biodiversity and landscape. The alpine and subtropical climates of Sikkim attract tourists during summers and winters. It has ~ 0.65 million permanent residents and is one of India’s pioneering regions for TB control. The state has one of the highest TB notifications in the country ranging from 260 to 300 per 100,000 population per year, which is comparable to major urban cities in India. Sikkim is also home to several Buddhist monasteries where students/monks from diverse and faraway places visit. These monks are frequent travellers and therefore happen to be at risk of TB exposure, which makes them potential transmitters of disease outside of the monasteries. In this study, we assessed the prevalence of ATB and TBI among monks of all age groups, using molecular/immunological screening to measure the risk factors associated with a congregate facility.

## Method

### Study site

The study is part of National TB Elimination Program (NTEP)-Sikkim conducted at Rumtek Monastery and Institute for higher Buddhist study (Sikkim, India). The monastery is currently the largest in Sikkim, and it is home to a community of 350 monks from the different states of India, Nepal and Bhutan. The Dharma Chakra Center of the monastery is the center of belief and conviction. Over five thousand national and international devotees and tourists annually visit the monastery.

### Study design

A cross-sectional study was carried out on the population of Rumtek, aiming to make the region “TB-free” as part of the broader goal of achieving “TB-free Sikkim”. The main objective was to conduct on-site screenings for ATB and LTBI.

### Data collection and procedure

The active case finding (ACF) for the TB screening program was carried out from September 2018 to November 2019 at the Tso-Jey clinic, located in the monastery premises. The sample of the study includes 174 participants who have met the inclusion criteria such as any TB symptoms, history of TB contacts, history of TB and ≥9 years of age. Exclusion criteria on the basis of participants who are under the age of 9 years, not willing to be part of the study and result discrepancy due to delay in sample transportation. The students, teachers and staff of various age groups (9–73 Yrs.) were invited for clinical screening at Tso-Jey clinic by trained healthcare workers from the National TB Elimination Program (NTEP)-Sikkim. Information was collected on TB-related symptoms including cough, fever, night sweats, weight loss, fatigue and history of TB and nutritional assessment. Data was also collected on socio-demographics and household characteristics, family history of TB, alcohol and smoking habits.

To study the prevalence of TBI among the inhabitants, assuming a global TBI prevalence of 24.8% [10], an estimated sample size of 172 with 10% dropout was calculated to achieve a 95% confidence level with a precision of 5%. Participants were invited to participate in the LTBI screening. Consent forms were filled by the willing participants (residents of the monastery). Guardian consent, along with verbal assent, was obtained from minors. Information was collected on dietary intake, home visit schedule, household contact history, and Bacillus Calmette-Guérin (BCG) vaccination (BCG scar mark was searched).

All the participants and samples collected in the study were coded so that the information about the participant could only be accessed by principal investigators of the project; the identity of the subjects is kept confidential for any presentations or publications resulting from the study.

### Ethics approval and consent to participate

The study was approved by the institutional ethical committee of SIR THUTOB NAMGYAL MEMORIAL (STNM) Hospital, Gangtok, Sikkim (05/IEC/STNM/16, dated 23-12-2016). Considering the study to be an active case screening program, all residents of the monastery were eligible for TB symptom screening and testing. The project was explained to each participant in vernacular language; and written consent was obtained to participate in the study as well as to publish the results, from each participant. All methods were performed in accordance with the relevant guidelines and regulations, research work involving human blood samples was performed in accordance with the Declaration of Helsinki.

### Sample processing and laboratory analysis for active TB (ATB) screening

For onsite ATB screening, a real-time micro-PCR-based TrueNat MTB (Molbio Diagnostic Pvt. Ltd. India) system was installed at the Tso-Jey clinic, and hands-on training was provided to the technicians. Spot sputum samples were collected from the study participants among the students and teachers of the Rumtek monastery, and tested on the TrueNat platform by a trained technician as per the manufacturer’s guidelines. To rule out active TB in TBI-positive participants, early morning sputum samples were collected and transported to the Intermediate Reference Laboratory, New STNM Hospital, Gangtok, Sikkim, in cold chain and GeneXpert MTB/Rif (Cephid, USA) test was performed as per the manufacturer’s guidelines.

### Sample processing and laboratory analysis to detect TB infection (TBI)

A sample of 4 ml of whole blood sample was collected in lithium heparin tubes from each consented participant by skilled project staff, and transported to the Intermediate Reference Laboratory, New STNM Hospital, at 4–8^°^C (not beyond 48 hours). Interferon-gamma release assay (IGRA) was carried out as per the manufacturer’s guidelines. Briefly, 1 ml of blood was added to each of the four QFT-plus (Qiagen, Germany) antigen tubes; tubes were incubated at 37ºC for 16–20 hours and subsequently centrifuged at 2500 rpm for 15 minutes. The released IFN-γ concentration was measured using an enzyme-linked immunosorbent assay. A sample was considered positive (TBI) if the value of the TB1 or TB2 minus Nil control was > 0.35 IU/ml as per the manufacturer’s guidelines.

### Monitoring and follow-up of IGRA-positive individuals

TBI-positive individuals were followed for one year through phone calls, personal meetings, outpatient medical record review and quarterly review for any TB symptoms; TrueNat MTB testing was done if clinical symptoms were detected at Tso-Jey clinic.

### Statistical analysis

Data entry and analysis were done using *IBM (SPSS)* version 26. Categorical variable were presented as frequency distribution and percentages, and continuous data were presented as the interquartile range (IQR). Poisons Chi-square test was performed to compare the frequencies of characteristics of participants with or without TBI (IGRA positive or negative respectively). Univariate logistic regression was used to identify factors associated with TBI. Factors showing a significant influence in univariate analysis were included in the multivariable logistic regression model. Starting from a full model with all significant factors, we employed a backward Wald elimination approach removing the least significant factor one at a time until the remaining factors were significant. Association between predictors and TBI are summarized in odds ratio (OR) along with a 95% confidence interval (CI), where a *p*-value <0.05 was considered significant. Gender and ethnicity being important factors were added in the model.

## Results

### Study participants

**Fig 1:**
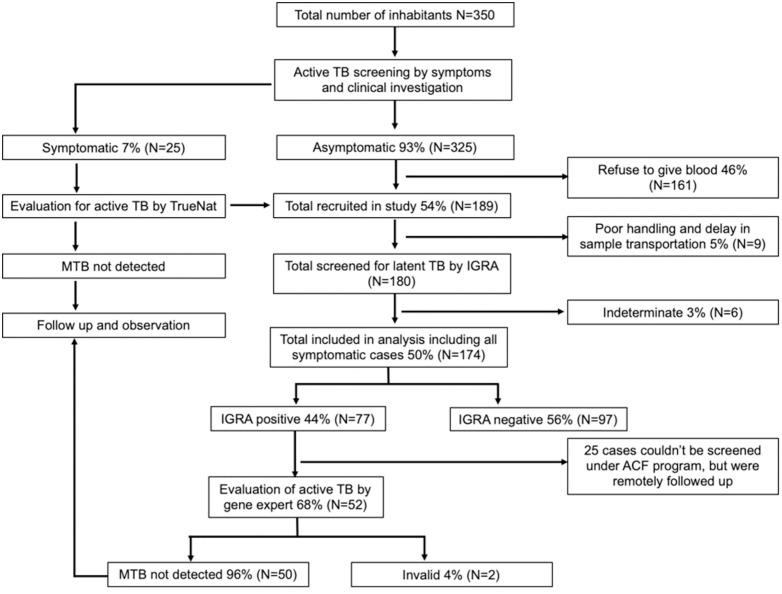
Flow diagram showing study plan and number of samples screened/identified in each group by IGRA screening.

At the time of our study, among the 350 residents of Rumtek monastery, 54% (189/350) agreed to participate in the TB/TBI screening study (Fig 1); 15 out of these189 participants were excluded due to indeterminate/unavailability of QFT plus test results (six indeterminate, nine poor handling and delay in transportation). Complete QFT plus results of 174 participants were obtained and included in the analysis. The overall prevalence of TBI among the participants at Rumtek Monastery was 44.2% (77/174).

Among the 174 participants (age range 9–73 years, median age 21), 166 (95.4%) were male. Majority of participants, 65.2% (107/174), were between the 15–35 age group, followed by less than <14 years age group 20.7% (34/174). 12.8% (22/174) participants had a history of TB, whereas about 2.9% (5/174) had a family history of TB. The BMI ranged from 16.5 to 58.4 (median 24.19, interquartile range 20–28). Students accounted for 75.9% (132/174) of the study cohort, followed by 12.1% (21/174) of teachers and 12.1% (21/174) of monastery staff. A total of 36.8% (64/174) participants used to visit their homes occasionally, 35.1% (61/174) yearly and 28.2% (49/174) on a weekly basis. A total of 49.4% (86/174) of participants had BCG scars. The largest proportion of participants, 63.8% (111/174), ethnically belonged to Sikkim. The TBI in male population is 45.2%, which is higher in comparison to females (25%), with statistically significant difference (*p* = 0.010), which could be due to smoking habits etc. [11,12].

**Fig 2:**
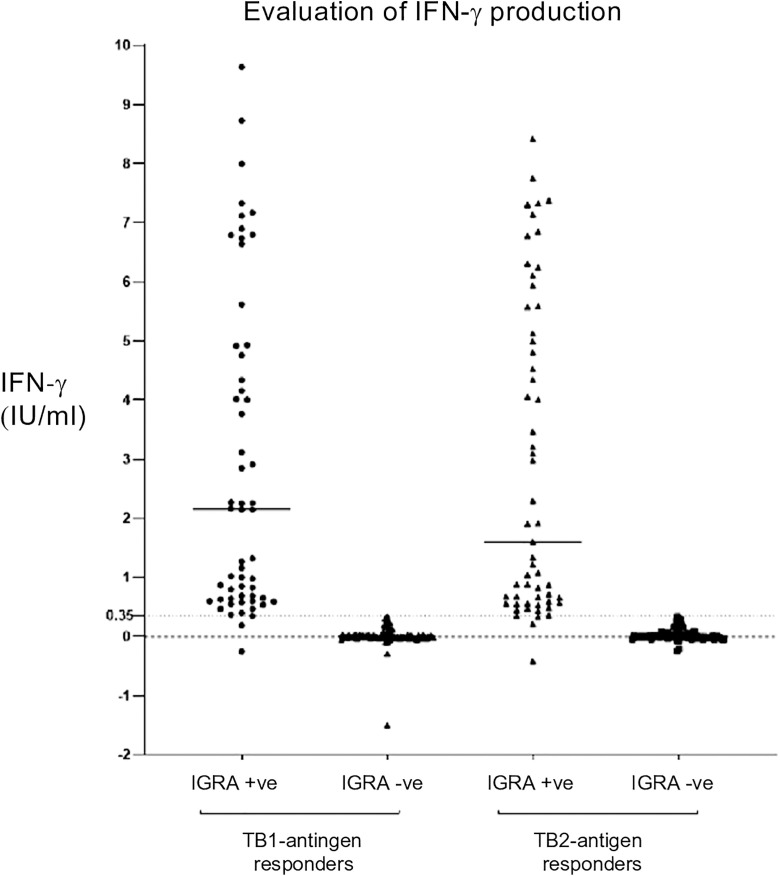
Comparison of characteristics between IGRA positive and IGRA negative participants.

A higher TB2 antigen response (TB2-TB1 > 0.6 IU/ml) was observed in 11 (~14%) of TBI subjects (Fig 2).

### Active TB screening of symptomatic and TBI positive participants

Out of 350 inhabitants, ~ 7% (25/350) showed TB-related symptoms, and ~93% (325/350) were asymptomatic. A total of 189 participants, including symptomatic cases, agreed to participate in the study. Onsite screening of sputum samples of symptomatic TB-suspected individuals was carried out using the TrueNat MTB platform at Tso-Jey clinic, however no active TB case was detected in this set. TBI analysis was carried out for 189 participants among the 350 inhabitants of the monastery, however 15 participants were excluded from the study due to result discrepancies. The IGRA analysis of total 174 participants showed prevalence of TBI as 44.2% (77/174). Sputum samples of 68% (52/77) these IGRA-positive cases were screened using the GeneXpert test, however no case of active TB was detected. Samples from 33% (25/77) of IGRA-positive individuals could not be collected as they returned back to their native place after completing their studies at the monastery. All the TBI positive subjects were followed-up for one year through telephone calls, however, none of TBI positive individual reported TB related symptom and sign during one-year follow-up period.

### Comparison of characteristics between IGRA Positive and IGRA Negative participants

The overall prevalence of TBI at Rumtek Monastery was 44.2% (77/174). We compared frequencies of characteristics between IGRA positive and IGRA negative participants (Table 1). TBI rates increased with age: < 14 years, 23.5%; 15–35 years, 45.3%; > 36 years, 69.6%, and this positivity trend was significant (*p* = 0.003). The presence of other factors such as ethnicity, gender and BCG scar showed no significant correlation with the TBI cases (*p* = 0.281, *p* = 0.262, *p* = 0.553 respectively). Participants with TBI showed a significant correlation with abnormal BMI (underweight, overweight and obese) >25- < 18.5: 59.8% (46/86) (*p* = 0.010). The positivity rate among the participants with having past history of TB, was immensely higher at 81.8% (18/22), similarly, all participants, that have been in household contacts of TB cases, were found to be TBI positive (5/5) (*p* = 0.011). Differences in occupation and travelling factors were also significant (*p*=<0.001) and (*p* = 0.031) respectively, with higher TBI rates among participants travelling more frequently; on weekly basis, 59.2% (29/49), followed by 42.2% (27/64) for occasional travel, and 34.4% (21/61) yearly travel. The TBI rate was comparatively higher in teachers 81% (17/21) followed by office staff 52.4% (11/21) and 37.1% (49/132) students.

**Table 1:** Categorical variable information and comparison of characteristics between IGRA positive and IGRA negative individuals.

Variables		No. or Median	Percent or (IQR)	IGRA Negative N (%)	IGRA Positive N (%)	*p* value by χ2 test
Gender	Female	8	4.6	6(75)	2(25)	0.262
	Male	166	95.4	91(54.8)	75(45.2)	
Age Median(IQR)	21 (16-30)			18(14-25)	28(20-34)	
	<14 Years	34	20.7	26(76.5)	8(23.5)	0.003
	15-35Years	107	65.2	64(54.7)	53(45.3)	
	>36 Years	23	14.0	7(30.4)	16(69.6)	
Family History of TB	No	169	97.1	97(57.4)	72(42.6)	0.011
	Yes	5	2.9	0(0.0)	5(100)	
BMI (Kg/m2) Median (IQR) Normal Abnormal	24.19 (20-28)			22(20-25)	25(22-29)	
	18.5-25	89	51.1	58(65.2)	31(34.8)	0.010
	>25-<18.5	85	48.9	39(40.2)	46(59.8)	
History of TB	No	152	87.4	93(61.2)	59(38.8)	0.001
	Yes	22	12.8	4(18.2)	18(81.8)	
Traveling	Weekly	49	28.2	20(40.8)	29(59.2)	0.031
	Occasionally	64	36.8	37(57.8)	27(42.2)	
	Yearly	61	35.1	40(65.6)	21(34.4)	
Occupation Type	Student	132	75.9	83(62.9)	49(37.1)	< 0.001
	Teacher	21	12.1	4(19.0)	17(81.0)	
	Office staff	21	12.1	10(47.6)	11(52.4)	
BCG	Yes	86	49.4	46(53.5)	40(46.5)	0.553
	Unknown	88	50.6	51(51.6)	37(49.3)	
Ethnicity	Sikkim	111	63.8	57(51.4)	54(48.6)	0.281
	Assam	14	8	9(57.1)	5(42.9)	
	Nepal	25	14.4	19(76)	6(24)	
	Bhutan	13	7.5	7(53.8)	6(46.2)	
	Others	12	6.3	6(54.5)	5(45.5)	

IQR: interquartile range; **χ2:** Pearson’s chi square test, BMI: body mass index; TB, Tuberculosis, BCG, Bacillus Calmette Guerin.

### Factors associated with TBI

Univariate regression analysis (Table 2) revealed several factors significantly associated with an increased likelihood of TBI. The odds ratios (ORs) for age (OR=7.42, 95% CI = 2.25–24.42, *p* < 0.05), BMI (OR=2.20, 95% CI = 1.19–4.06, *p* < 0.05), a history of tuberculosis (TB) (OR=7.09, 95% CI = 2.28–21.99, *p* < 0.05), occupation (OR=7.19, 95% CI = 2.29–22.62, *p* < 0.05), and frequent travel (OR=2.76, 95% CI = 1.27–6.0, *p* < 0.05) all indicated a statistically significant positive association with TBI. Specifically, the probability of TBI was higher for teachers (OR=7.19, 95% CI = 2.29–22.62) compared to staff members (OR=1.86, 95% CI = 0.73–4.70). Similarly, participants who travelled weekly (OR=2.76, 95% CI = 1.27–6.0) were at a significantly greater risk of TBI than those who travelled occasionally (OR=1.39, 95% CI = 0.67–2.86).

**Table 2:** Univariate and Multivariate regression analysis of risk factors predicting TBI.

Variables		Risk of TBI (Univariate analysis) OR 95% C I	*p* value	Risk of TBI (Multivariate analysis) AOR 95% C I	*p* value
Age Median(IQR)	≤14 Years	Ref		Ref	
	15 - 35Years	2.69 (1.12 - 6.43)	0.026	2.15(0.88 - 5.25)	0.093
	≥ 36 Years	7.42 (2.25 - 24.42)	0.001	4.69(1.35 −16.23)	0.015
Family History of TB#	No	Ref			
	Yes	NA		NA	
BMI(Kg/m2) Median(IQR)	18.5 - 25	Ref		Ref	
	≤18.5 - ≥ 25	2.20 (1.19 - 4.06)	0.011	1.87 (0.97 −3.59)	0.058
History of TB	No	Ref		Ref	
	Yes	7.09 (2.28 −21.99)	0.001	5.38(1.679-17.28)	0.005
Occupation Type	Student	Ref			
	Teacher	7.19 (2.29 −22.62)	0.001		
	Office staff	1.86 (0.73 - 4.70)	0.188		
Travelling	Weekly	2.76 (1.27 - 6.0)	0.01		
	Occasionally	1.39 (0.67 - 2.86)	0.373		
	Yearly	Ref			
Ethnicity	Sikkim	Ref			
	Assam	0.79 (0.25 - 2.43)	0.683		
	Nepali	0.33 (0.12 - 0.89)	0.030		
	Bhutan	0.90 (0.28 - 2.86)	0.865		
	Others	0.88 (0.25 - 3.05)	0.840		

# Odds not available due to missing value in IGRA negative column. OD- Odds ratio, AOR-Adjusted Odds Ratio, CI- Confidence Interval, IQR- Interquartile Range, BMI- Body mass index, Ref- Reference.

In multivariate logistic regression analysis, age, BMI, and a history of TB were strongly associated with TBI. Specifically, the odds of TBI increased significantly with age. For the 15–35 age group, the adjusted odds ratio (AOR) was 2.15 (95% CI = 0.88–5.25), while for those over 36 years old, the AOR was 4.69 (95% CI = 1.35–16.23), compared to the reference group under 14 years of age. Additionally, participants with household contact with TB cases, exhibited a notably higher TBI probability, with 100% of those exposed testing positive (5/5), marking this as the most significant infection-related risk factor for TBI. Table 2 outlines the probabilities of TBI in participants with abnormal BMI (≤18.5 or ≥25) (AOR = 1.87, 95% CI = 0.97–3.59), which was also positively correlated with TBI in univariate analysis and showed near-significant results in the multivariate analysis.

## Discussion

The study is the first-ever study in Sikkim for any congregate living facility to describe the burden of TB as well as the risks of TBI. The cross-sectional study found that the overall TBI rate was 44.2% which is comparable to an earlier study [13], prevalence of TBI by QFT-G was 48% in poor ventilated high TB endemic zone in India and also comparable to the studies in other developing countries [13–16]. Another study showed 18% TBI among Tibetan school children whereas 53% of adult staff was TBI positive [17]_._ Prevalence is notably higher than general population and is comparable to an earlier study [13], reported 48% prevalence of TBI by QFT-G in poor ventilated high TB endemic zone in India and also comparable to the studies in other developing countries [13–16]. High prevalence in present study is possibly due to the congregate living conditions, frequent travel history of residents, and exposure to ATB individuals, which are less prevalent in general or outpatient populations. Further, population type being studied and the choice of method employed for TBI testing also influence the prevalence and reliability of estimates. Our study used QFT-G for TBI detection, which offers higher specificity than TST [18]. In addition, TrueNat and GeneXpert systems were used to ensure that there is no undiagnosed ATB among TBI cases, which is a limitation in other studies using only hospital-based retrospective data, which excluded asymptomatic individuals. The present study targeted a culturally distinct, secluded population and a proactive screening approach to provide a more accurate picture of TBI burden and population-specific risk factors in a congregate living setting.

Similar study on Tibetan schools showed 18% and 53% TBI among school children and adult staff, respectively, which was slightly higher than our study [17]. A meta-analysis estimated prevalence TBI in India to be 36% in general population excluding high risk group and 41% in community-based cohort studies in the population with high- risk groups [19]; comparatively higher prevalence of TBI in our study, highlights the burden of active TB cases within the region and is comparable to TBI in high risk group population such as health care workers in high burden countries like Indonesia (44.1%) [20,21]. In the broader Southeast Asian context, TBI prevalence varies widely due to differences in TB incidence, population risk factors and diagnostic practices [22] Studies from countries like Vietnam, Indonesia, and the Philippines report TBI rates ranging from 30% to over 50% in high-risk groups, particularly in urban slums, prisons, and healthcare settings [23], which also correlates with our study. Factors contributing to TBI in congregate facility is rather crucial to understand in terms of planning targeted intervention to reduce TBI. Communal living facilities differ from other rural and urban populations in terms of population mobility and living conditions. While socioeconomic status in these facilities may exceed that of urban slums, the presence of shared sleeping quarters, communal dining areas, and collective places of worship produces conditions comparable to known high‑risk groups such as prisoners and health‑care workers. These circumstances increase the likelihood of repeated exposure to active tuberculosis (ATB), thereby contributing to a higher prevalence of tuberculosis infection (TBI).

Univariate analysis of the risk factor for TBI in our study showed a significant association with age, BMI, occupation and frequent travelling, multivariate regression analysis with these factors, age and BMI appeared strong predictor. Older age is significantly associated with a higher likelihood of TBI (*p* < 0.05). This aligns with previous research indicating that the immune system’s ability to contain latent infections diminishes with age, making older individuals more susceptible to TBI [24]. Multivariate regression analysis with these factors, age and BMI appeared strong predictor. Past history of TB was also a strong predictor of positive IGRA, as expected in people with long-term health complications and weakened immune system with a history of TB [25,26], suggesting the need for monitoring and management to prevent reactivation of TBI, which can progress to TB. Immediate contacts having past history or experience of exposure to TB, within the congregate settings of the monastery, is one of the reason for high frequency of TBI in the study population. Risk of contracting TBI is significantly higher among the household contacts compared to the general population, due to prolonged and close exposure to ATB case [27–29]. The detection rate of TBI among family contacts of TB patient ranged from 0.2%−2% in high-income countries [30,31]. In a prospective follow-up study (2008–2012), in a high TB burden setting in India, 76 out of 1511 household contacts developed active TB [32]. The present study is based on inhabitants of Monastery where monks live together, although they come from different countries like Tibet, Nepal, Myanmar, etc. In this study, no TB breakdown occurred during the follow-up for a span of one year. A short one-year follow-up is a limitation of our study when assessing long-term follow-up of TBI reactivation and progression to ATB, longer follow-up period is essential to accurately capture reactivation events. Conversion of TBI to active TB was reported to be 5–10% by WHO [24], over their lifetime, with the highest risk occurring within the first two years after infection [33].

Several factors were identified as significant contributors to the risk of TBI in our study. Advancing age was a key factor, with older individuals exhibiting higher rates of TBI, immune system’s tends to weaken with age, making it less effective to contain latent infections, that increases exposure time making older individuals more susceptible to TBI, finding supported by study on health care worker and elderly population in Indonesia and China [34,35]. Similar findings have been reported in studies from TB-endemic regions in India, where the risk of TB infection increases with age [13]. We also found that travel frequency was a potential risk factor for acquisition of TB infection, as it elevated the likelihood of exposure to TB. Participants who frequently travelled to their native places had a higher rate of IGRA positivity, compared to those who travelled only once a year. This suggests that frequent travel increases the risk of encountering TB infection, due to increased contact with diverse populations and environments both during travel and in the travellers’ home regions. A meta-analysis estimated higher prevalence Traveling from low TB incidence countries to intermediate or high TB incidence countries had higher TBI, also highlighted that healthcare workers and military personnel, who frequently travel, had the highest cumulative incidence of TBI [36]. Moreover, monastery teachers exhibited a higher rate of TBI, likely due to prolonged, close indoor exposure in classrooms, where larger groups of students regularly visit the monastery. In addition, body mass index (BMI) emerged as a significant confounding factor in our study. It is well-established that both underweight and obese individuals are at greater risk of contracting tuberculosis (TB), with considerable evidence linking malnutrition as a key determinant of TB susceptibility [37,38]. Despite extensive research, the precise relationship between BMI and TB infection remains incompletely understood, particularly in relation to active TB disease [39,40]. In a population-based, multi-center study conducted in rural China, BMI was independently associated with increased susceptibility to TB; notably, this association was stronger in areas with a high prevalence of TB [41]. It has also been suggested that excessive adiposity may impair immune function and host defence in obese individuals, potentially increasing their vulnerability to infections including TB [42,43].

The present study reported a higher TB2-TB1 difference (>0.6 IU/ml) in 14% of TBI participants. It has been shown that TB-2 specific response elicited by CD8 + T cells are associated with active TB, both among immuno-compromised and healthy individuals [44–47]. However, in our study all such TBI cases were asymptomatic during screening and follow-up; a higher TB2 response (CD4 and CD8) among these individuals could probably be due to recent exposure to active TB cases during travelling or at their native place. Longer-term follow-up is needed to determine if the reported CD8 + T cell response is a biomarker for impending TB or reactivation.

In this study, no cases of active TB or disease progression were detected during the one-year follow-up using PCR methods. However, 12.8% (22/174) of participants reported a history of past TB. If this self-reported data is accurate, the estimated incidence would exceed 12,000 cases per 100,000 population, which is nearly 40 times higher than the incidence rates observed in Sikkim and other Indian cities. Earlier, studies have shown that reactivation of TB occurs in a small fraction of cases (~5%−15%), typically within the first 2–5 years following initial infection [48,49]. While the exact causes of TB reactivation remain incompletely understood, it is believed to result from a combination of bacterial, host, and environmental factors [50]. In a large, population-based prospective cohort study in the UK, the positive predictive values of the interferon-gamma release assay (IGRA) and tuberculin skin test (TST) were found to be only 3%−4%, meaning that only 3% to 4% of individuals with positive TBI results progressed to active TB over a median follow-up period of 2.9 years [51]. However, it’s important to note that findings from low-incidence studies may not be directly applicable to high-burden settings, where the dynamics of TB reactivation can differ significantly. Various comorbidities and risk factors are known to increase the likelihood of TB reactivation, leading to higher rates of active TB development. Therefore, conditions associated with TBI reactivation should be categorized based on their risk level (high, moderate, slight, low, or very low), and managed accordingly, with preventive TB therapy and ongoing monitoring for TB outbreaks [52]. The need for comprehensive TB and TBI screening within the monastery setting is further supported by the elevated risk of exposure, particularly among children, adults in high-risk occupations (e.g., teachers), and individuals with medical conditions that increase the risk of progression to active TB. The interaction of monastics with the general public, whether through spiritual communion or frequent visits to their home regions, intensifies the bilateral risk of TB exposure and infection, making it particularly critical for those living in and interacting with the monastery.

Several key limitations affected this study’s scope and findings. First, the relatively small number of participants with TBI and the brief one-year follow-up period limited our ability to fully assess TB reactivation rates. The short follow-up was unavoidable, as most monks typically return to their home regions after completing their education. PCR-based detection presented another significant limitation. Both TrueNat MTB and GenXpert MTB/Rif systems, while comparable in performance, show reduced sensitivity in cases with low bacterial loads. A recent multi-center study demonstrated that TrueNat MTB and TrueNat MTB Plus achieved sensitivities of only 36% and 47% respectively in smear-negative, culture-positive sputum specimens [53]. Given that our study participants were largely asymptomatic, suggesting potentially low bacterial burden if TB was present, relying on PCR testing of a single sputum sample may have been insufficient for accurate detection. Although it is technically constraints for remote settings, the study recommends a more comprehensive screening approach that combines chest radiography and sputum culture with symptom assessment and PCR testing. This multi-modal strategy would likely improve case detection rates and provide more robust findings. The current study recommends implementation of comprehensive TB control strategies tailored to congregate settings like monasteries, i.e., baseline testing for new entrants, routine TBI screening and contact tracing in cases of ATB. Initiating recommended TPT regimens for treating TBI cases [54], improving ventilation in communal settings [55] and health education and awareness sessions on TB symptoms and its transmission.

## Conclusion

This study aimed to assess the prevalence and risk factors associated with latent tuberculosis infection (LTBI) among residents of the Rumtek Monastery in Sikkim, a high-risk congregate setting. Monasteries serve as vital spiritual centers, deeply woven into their communities’ fabric through festivals, ceremonies, and special events. These sacred spaces house monks across all age groups, including vulnerable populations such as children under 15. The combination of communal living, frequent public interaction, and the mobile nature of monastic life creates unique challenges for TB prevention. The findings revealed a significant association between TBI and several factors, including increasing age, abnormal BMI, a history of tuberculosis, occupation (particularly being a teacher), and frequent travel. These results underscore the heightened vulnerability of individuals in monastic environments due to close living quarters, communal activities, and frequent mobility. These findings present a compelling case for the National TB Elimination Program (NTEP) to develop targeted interventions for monasteries and similar congregate settings. Such interventions should focus on optimizing active TB diagnosis, TBI screening, and implementing comprehensive prevention strategies. Currently available preventive measures, including the combined treatment with Isoniazid and Rifapentine (INH-RPT), could prove particularly valuable in these settings. This aligns with NTEP’s broader strategy of focusing on high-risk populations in congregate settings to prevent TBI progression to active disease.
